# Retraction: Naloxone’s Pentapeptide Binding Site on Filamin A Blocks Mu Opioid Receptor–Gs Coupling and CREB Activation of Acute Morphine

**DOI:** 10.1371/journal.pone.0266629

**Published:** 2022-03-30

**Authors:** The PLOS ONE Editors

Following the publication of this article [1], concerns were raised regarding results presented in Figs 1, 2 and 5. Specifically,

- In the right MOR panel of Fig 1A, the density of background noise just below the results presented in lanes 1–2, and the density of background noise immediately surrounding the band in lane 4 do not appear to match the background density elsewhere in the blot.
- In the third MOR panel on the left side of Fig 2A, the VAAGL + Morphine 60 Min result appears to be missing from the panel.
- In the third MOR panel on the right side of Fig 2A, when levels are adjusted, there appears to be a vertical irregularity suggestive of a splice line between the results presented in lanes 6–7.
- In the third pS^133^CREB panel of Fig 5A, when levels are adjusted, there appear to be vertical irregularities suggestive of splice lines between the results presented in lanes 1–2 and lanes 6–7.

The first author confirmed that the VAAGL + Morphine 60 Min result was inadvertently cropped out of the published Fig 2A MOR panel and provided a replacement figure. However, the first author disagreed with the concerns raised with the Fig 1A and Fig 2A MOR panels and the Fig 5A pS^133^CREB panel, stating that each panel was obtained from a single blot and the observed irregularities were likely due to image compression artifacts.

The first author provided image data to support the contested western blot results in this [1] and other PLOS ONE articles [2–5]. Per PLOS’ assessment of the data files, the pixel patterns in background areas of blot images provided for multiple panels in [1–5] appear more similar than would be expected for data obtained in independent experiments. The first author stated that the repetitive features in the background noise of the underlying data are likely the result of scanner artifacts.

During PLOS’ assessment of the underlying data, it was noted that neither the published panels nor the image data for the Fig 2A middle left MOR panel included a positive control. The absence of a positive control to confirm a successful western blot calls into question the reliability of results presented in this panels. In addition, the editorial assessment of the image data provided for the Fig 5A middle pS^133^CREB panel did not match the panel presented in the published figure.

The data and comments provided to PLOS did not resolve the concerns about the integrity and reliability of the reported data. In light of these issues, the *PLOS ONE* Editors retract this article.

The authors did not agree with the retraction. HYW stands by the article’s findings.
